# Factors driving the halophyte rhizosphere bacterial communities in coastal salt marshes

**DOI:** 10.3389/fmicb.2023.1127958

**Published:** 2023-02-22

**Authors:** Rumiao Wang, Lijuan Cui, Jing Li, Wei Li

**Affiliations:** Institute of Wetland Research, Chinese Academy of Forestry, Beijing Key Laboratory of Wetland Ecological Function and Restoration, Beijing, China

**Keywords:** coastal wetland, salt marsh, bacterial community, soil metabolomics, coastal halophytes, rhizosphere

## Abstract

**Introduction:**

Root-associated microorganisms promote plant growth and provide protection from stresses. Halophytes are the fundamental components maintaining ecosystem functions of coastal salt marshes; however, it is not clear how their microbiome are structured across large spatial scales. Here, we investigated the rhizosphere bacterial communities of typical coastal halophyte species (*Phragmites australis* and *Suaeda salsa*) in temperate and subtropical salt marshes across 1,100 km in eastern China.

**Methods:**

The sampling sites were located from 30.33 to 40.90°N and 119.24 to 121.79°E across east China. A total of 36 plots were investigated in the Liaohe River Estuary, the Yellow River Estuary, Yancheng, and Hangzhou Bay in August 2020. We collected shoot, root, and rhizosphere soil samples. the number of pakchoi leaves, total fresh and dry weight of the seedlings was counted. The soil properties, plant functional traits, the genome sequencing, and metabolomics assay were detected.

**Results:**

The results showed that soil nutrients (total organic carbon, dissolved organic carbon, total nitrogen, soluble sugars, and organic acids) are high in the temperate marsh, while root exudates (measured by metabolite expressions) are significantly higher in the subtropical marsh. We observed higher bacterial alpha diversity, more complex network structure, and more negative connections in the temperate salt marsh, which suggested intense competition among bacterial groups. Variation partitioning analysis showed that climatic, edaphic, and root exudates had the greatest effects on the bacteria in the salt marsh, especially for abundant and moderate subcommunities. Random forest modeling further confirmed this but showed that plant species had a limited effect.

**Conclutions:**

Taken together, the results of this study revealed soil properties (chemical properties) and root exudates (metabolites) had the greatest influence on the bacterial community of salt marsh, especially for abundant and moderate taxa. Our results provided novel insights into the biogeography of halophyte microbiome in coastal wetlands and can be beneficial for policymakers in decision-making on the management of coastal wetlands.

## 1. Introduction

Coastal wetlands are the transition zones between terrestrial and marine ecosystems; they have high biodiversity and productivity, which provide multiple ecological services (Wang et al., 2021). Specifically, the intertidal salt marshes in temperate and subtropical areas are highly productive. The microbial community in the coastal wetland environment mediates the active biogeochemical process and maintains the stability and health of the coastal wetland system. The study of wetland microbial biogeography offers insights into mechanisms that generate and maintain wetland biodiversity, as well as the ecological services of coastal wetlands. A study of global wetlands showed that temperate latitude wetland soils harbored the highest bacterial diversity (Bahram et al., 2022), and it was reported that bacterial community composition was strongly correlated with latitude along the eastern coast of the United States (Blum et al., 2004). Multiple environmental factors including climate, soil properties, and vegetation types can affect the diversity and composition of bacterial communities and thereafter regulate microbial biogeography (Zhou et al., 2016; Gao et al., 2022; Yang L. et al., 2022; Yang Y. et al., 2022). However, after accounting for the host selection effect of halophyte plants (i.e., microbes can be strongly affected by the root exudates of plants in the rhizosphere), it is not clear which is the main driver of microbial biogeography in salt marshes.

The rhizosphere is a hotspot of organic matter transformation and plant–microbiome interactions (Bowen et al., 2017; Kuppardt et al., 2018; Tian et al., 2020). It is often assumed that the organic matter that is cycled by soil microbes is a complex mixture of metabolites that can be explored *via* soil metabolomics. Soil microorganisms can directly utilize rhizosphere soil metabolites as carbon sources, especially low-molecular weight organic compounds, which are derived from plant root exudates, microbial metabolites, and decomposition of plant, microbial, and soil organic matter (Dennis et al., 2010; Zhalnina et al., 2018). These soil metabolites have a strong impact on the microbial community structure, and vice versa (Swenson et al., 2018). Climate, season, vegetative, and reproductive growth phases of plants, anthropogenic disturbances, and regional factors can affect soil metabolites (Walker et al., 2003; Marquès et al., 2016; Swenson et al., 2018; Nguyen et al., 2020). However, soil metabolisms were mostly studied through laboratory-controlled experiments, rather than natural experiments (Zhao et al., 2019; Li et al., 2020; Song et al., 2020). To the best of our knowledge, we did not find any reports on the relationship between rhizosphere microbial communities and soil metabolism in natural salt marshes.

The objective of this study was to investigate the factors driving halophyte rhizosphere microbial communities across large spatial scales. We collected samples from temperate and subtropical salt marshes in east China across the Qinling Mountain–Huaihe River line (around 32–34° N in the eastern of China), which was a critical boundary for temperature, rainfall, and vegetation of China, separating southern and northern China (Cai et al., 2020). Soil physical and chemical properties, plant functional traits, and soil metabolism characteristics were measured; halophyte rhizosphere bacterial communities were characterized using the high-throughput amplicon sequencing approach.

## 2. Materials and methods

### 2.1. Study area and soil sampling

The sampling sites were located from 30.33 to 40.90°N and 119.24 to 121.79°E across east China (Supplementary Figure S1). The salt marshes in east China are dominated by *Phragmites australis* and *Suaeda salsa*. Soil samples were taken from the rhizosphere of different plants (*P. australis* and *S. salsa*). A total of 36 plots were investigated in the Liaohe River Estuary, the Yellow River Estuary, Yancheng, and Hangzhou Bay in August 2020. We collected shoot, root, and rhizosphere soil samples. The roots were collected by root auger (diameter 10 cm) to a depth of 30–40 cm, and the rhizosphere soil that was tightly attached to roots and rhizomes was brushed off and collected. The soil samples were stored at 4°C for physicochemical analysis, and subsamples stored at-80°C were used for further molecular and metabolite analyses.

**Figure 1:**
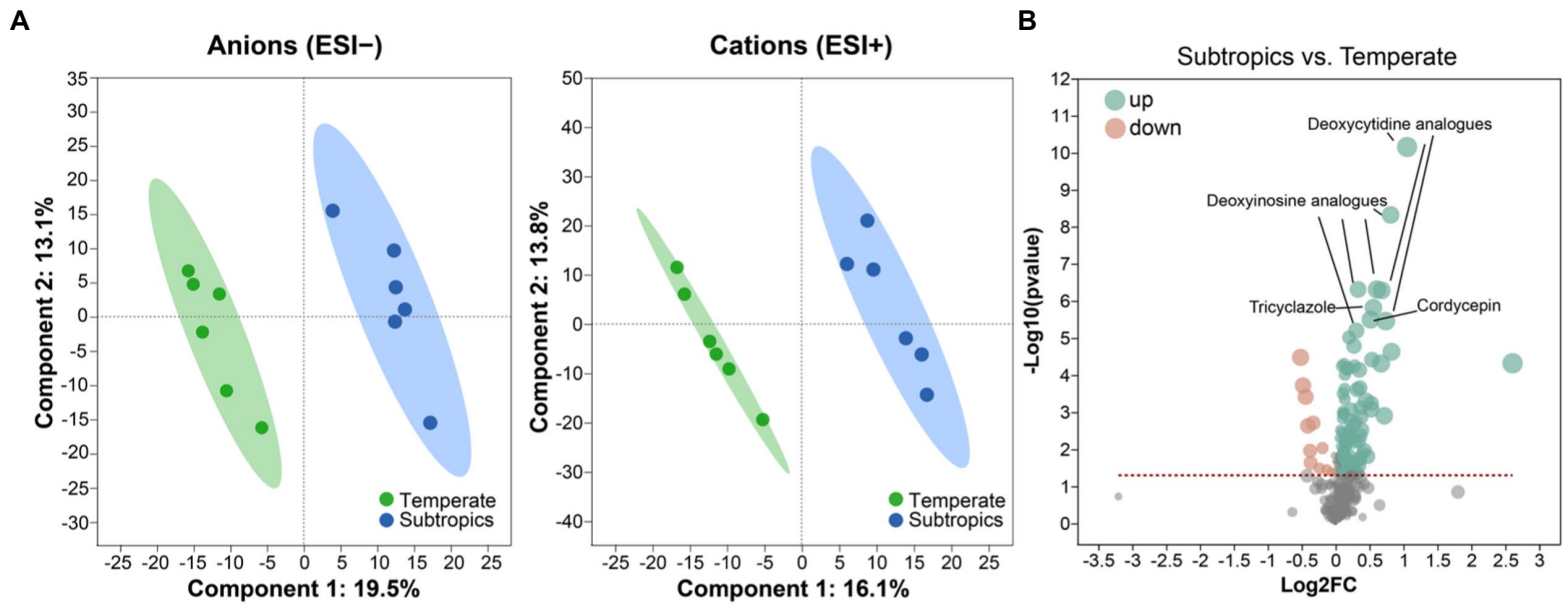
**(A)** Partial least squares discriminant analysis (PLS-DA) plots of the soil metabolites. **(B)** Volcano plot of differential metabolites between subtropics and temperate. Green dots represent a significantly upregulated expression when comparing subtropical to temperate samples; pink dots represent a significantly downregulated expression when comparing subtropical to temperate samples.

### 2.2. Soil and plant characterization

Soil total carbon (TC) and total nitrogen (TN) were determined by using an elemental analyzer (Elementar, Germany). Soil total organic carbon (TOC) and dissolved organic carbon (DOC) were measured by using a TOC analyzer (TOC-L CPH, Shimadzu). A pH monitor (Thermo, United States) was used to determine soil pH. Soil salinity was determined using a soil conductivity meter (LeiCi, China; Bennett, 1976). Soil soluble sugar and organic acids were determined *via* the anthrone colorimetric method and the ferric hydroxamate method using a spectrophotometer (Agilent Technologies, United States; Grandy et al., 2000; He et al., 2011). Soil metabolite analysis was determined using a UPLC-MS system (Thermo, United States). Both the positive-ion (ESI^+^) and negative-ion (ESI^−^) modes were used to determine the metabolite and annotated with KEGG and HMDB databases (Mönchgesang et al., 2016; Swenson et al., 2018). We used partial least squares discriminant analysis (PLS-DA) to compare the soil metabolite profiles between temperate and subtropics.

### 2.3. DNA extraction, high-throughput sequencing, and bioinformatics

Total soil DNA was extracted from 0.25 g of rhizosphere soil using a Powersoil® DNA Isolation kit (MoBio, United States). The 16S rRNA genes were PCR-amplified using the primers 338F/806R (Huws et al., 2007). The extracted DNA was measured using the Illumina MiSeq platform (Majorbio Bio-Pharm Technology Co., Ltd., Shanghai, China). The raw sequences were processed and analyzed *via* QIIME 2. Reads were end-trimmed to ensure a nucleotide quality score of >30 before merging, and operational taxonomic units (OTUs) were performed at a sequence identity of 97% (Edgar, 2010). The representative sequences were annotated with the SILVA database (Pruesse et al., 2007).

### 2.4. Statistical analysis

Bacterial OTUs with relative abundances greater than 0.1% were defined as abundant taxa; those with relative abundances below 0.01% were defined as rare taxa, and those with relative abundances between 0.01 and 0.1% were moderate OTUs (Jiao and Lu, 2020; Yang L. et al., 2022). After all data passed the preliminary Shapiro–Wilk test (*p* > 0.05), a *t*-test was used to assess the differences between the parameters (Hou et al., 2019). Partial least squares discriminant analysis (PLS-DA) with variable importance (VIP) values was used to identify the difference in soil metabolites (Darnaud et al., 2021). The PLS-DA model was validated with a permutation test (*n* = 200), *R*^2^ data are larger than *Q*^2^ data, and the intercept of Y and *Q*^2^ was less than 0 (*R*^2^ intercept = 0.9576, *Q*^2^ intercept = −0.3811), which means the model was fit and not overfitting (Chen et al., 2021). The alpha diversity indices including Chao1 and inverse Simpson index (or InvSimpson for short) were calculated by the R program package “vegan” (Oksanen et al., 2007). Nonmetric multidimensional scaling (NMDS) based on Bray–Curtis distance was conducted by the “vegan” package.

The network analysis was inferred using the SparCC-based algorithm Fastspar with a bootstrap procedure repeated 100 times, and only strong (*r* > 0.6) and significant (*p* < 0.01) correlations between OTUs were retained (Watts et al., 2019). The network nodes with high closeness (or betweenness) centrality value were identified as keystone hubs in the network: the network hubs were highly connected, both in general and within a module; the module hubs were highly connected within a module, the connectors provided links among multiple modules, and the peripherals had few links to other species (Poudel et al., 2016). Variation partitioning analysis (VPA) was used to assess the impact of environmental factors on bacterial communities using the “vegan” package (Wang et al., 2019). Before the performance of VPA, we assessed the collinearity of the variables by calculating the variance inflation factor (VIF). The factors were included in the VPA analyses, only when the collinearity VIF < 10 (Liao et al., 2019). The predictors of bacterial richness were identified *via* random forest modeling using the “randomForest” package in R (Liaw and Wiener, 2002). The importance of each predictor was determined by the increase in the mean square error (InMSE) and was averaged over 5,000 trees (Xu et al., 2018).

## 3. Results

### 3.1. Basic soil, plant, and soil metabolites properties

Generally, environmental factors showed significant differences between temperate and subtropical salt marshes (*t*-test; Table 1). The mean annual temperature (MAT), mean annual precipitation (MAP), daily temperature, and soil pH in the subtropical salt marsh were higher than that in the temperate salt marsh, while soil TN, TOC, DOC, soluble sugar, and organic acids were higher in temperate. The aboveground biomass was significantly higher in subtropical salt, while root biomasses and total root length revealed no significant differences between temperate and subtropical salt marshes (*t*-test; Table 2). A total of 442 metabolites were detected and identified among all soil samples. The metabolites were grouped as fatty acyls (20%), carboxylic acids and derivatives (13.33%), organooxygen compounds (11.52%), and prenol lipids (9.70%). Comprehensive analysis of all samples revealed a clear separation between temperate and subtropical salt marshes, in both the ESI^+^ and ESI^−^ data (Figure 1A). There were 107 metabolites that showed significant differences between the temperate and subtropical salt marshes (*p* < 0.05, VIP > 1; Figure 1B), and we found that the relative abundance of metabolites (including nucleoside analogs, pesticides, oxyinosine analogs, deoxycytidine analogs, tricyclazole, and cordycepin) in the subtropical salt marsh was significantly (*p* < 0.05) higher than in temperate one.

**Table 1:** Chemical properties of the soils in different groups.

	Temperate	Subtropics
Mean annual temperature (MAT; °C)	12.54 ± 10.83	17.10 ± 8.23^**^
Mean annual precipitation (MAP; mm)	382.5 ± 176.64	984.9 ± 370.66^**^
Daily temperature (°C)	28.2 ± 2.76	32.5 ± 1.05^**^
Soil pH	8.08 ± 0.31	9.18 ± 0.25^*^
Soil salinity (us/cm)	1,151 ± 550	1,189 ± 393
Soil TN (g/kg)	0.59 ± 0.24^*^	0.45 ± 0.32
Soil TOC (g/kg)	5.34 ± 2.15^*^	4.34 ± 3.82
Soil DOC (mg/kg)	1.36 ± 0.28^*^	0.89 ± 0.31
Soluble sugar (mg/kg)	4.84 ± 1.90^*^	3.82 ± 1.83
Organic acids (mg/kg)	16.11 ± 2.36^**^	5.66 ± 1.99

*Represent a significant difference at *p* < 0.05. **Represent significant difference at *p* < 0.01 (*t*-test).

**Table 2:** Variation in plant functional traits.

	*Phragmites australis*		*Suaeda salsa*	
	Temperate	Subtropics	Temperate	Subtropics
Aboveground biomass (g)	69.03 ± 10.01	195.05 ± 73.40^**^	64.95 ± 11.42	88.83 ± 13.67^**^
Roots biomass (g)	23.45 ± 9.80	26.86 ± 6.89	3.23 ± 2.76	6.59 ± 4.35
Total root length (cm)	30 ± 11.034	39.17 ± 7.89	15.5 ± 2.17	19 ± 8.58

** Represent significant difference at *p* < 0.01 (*t*-test).

### 3.2. Bacterial community composition in temperate and subtropical salt marshes

**Figure 2:**
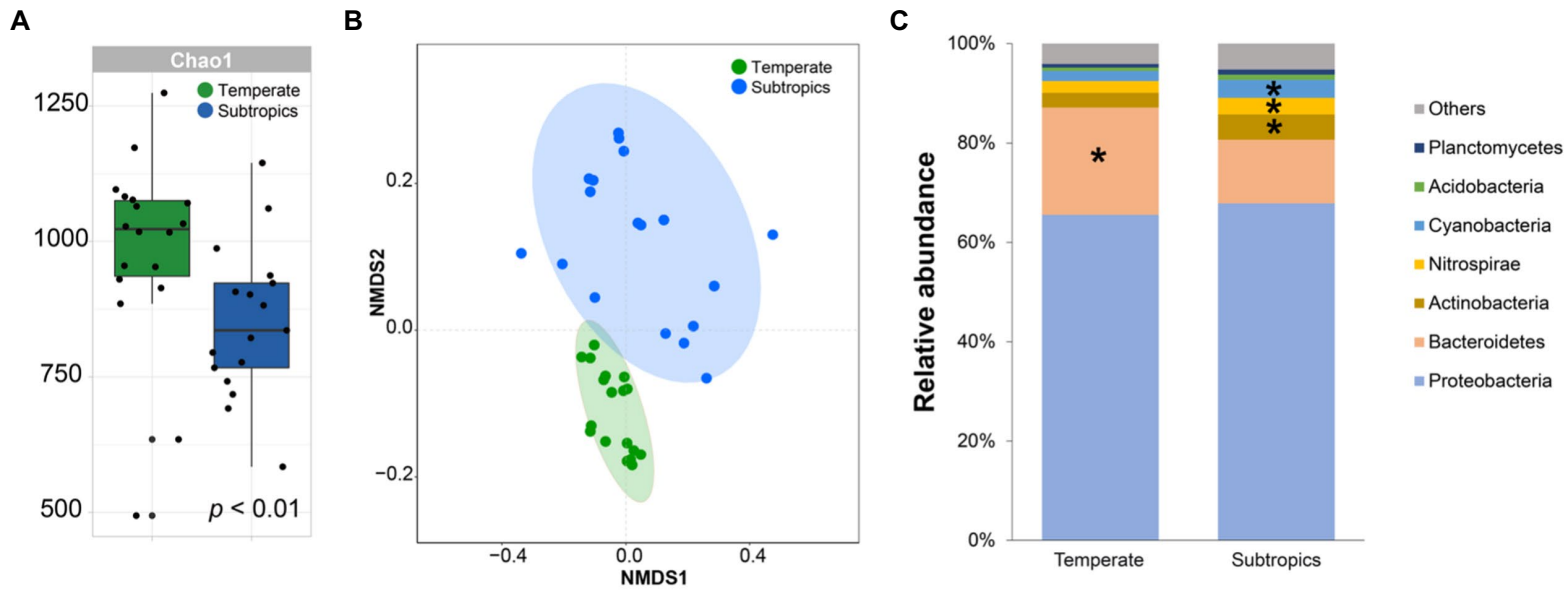
**(A)** Richness of bacteria in two groups; **(B)** NMDS analysis of bacteria; and **(C)** average relative abundances of bacterial phyla in two groups.

After quality filtering and the removal of chimeric sequences, 393,012 sequences were produced from the 36 samples. Finally, 26,266 OTUs were generated at 97% sequence identity. Bacterial communities in the temperate salt marsh were more diverse than in the subtropical salt marsh (Figure 2A). The overall composition of bacterial communities differed significantly between temperate and subtropics on the OTU level (Figure 2B). On the phylum level, the relative abundance of Bacteroidetes was significantly higher in temperate, Actinobacteria, Nitrospirae, and Cyanobacteria were significantly higher in the subtropical salt marsh (Figure 2C).

**Figure 3:**
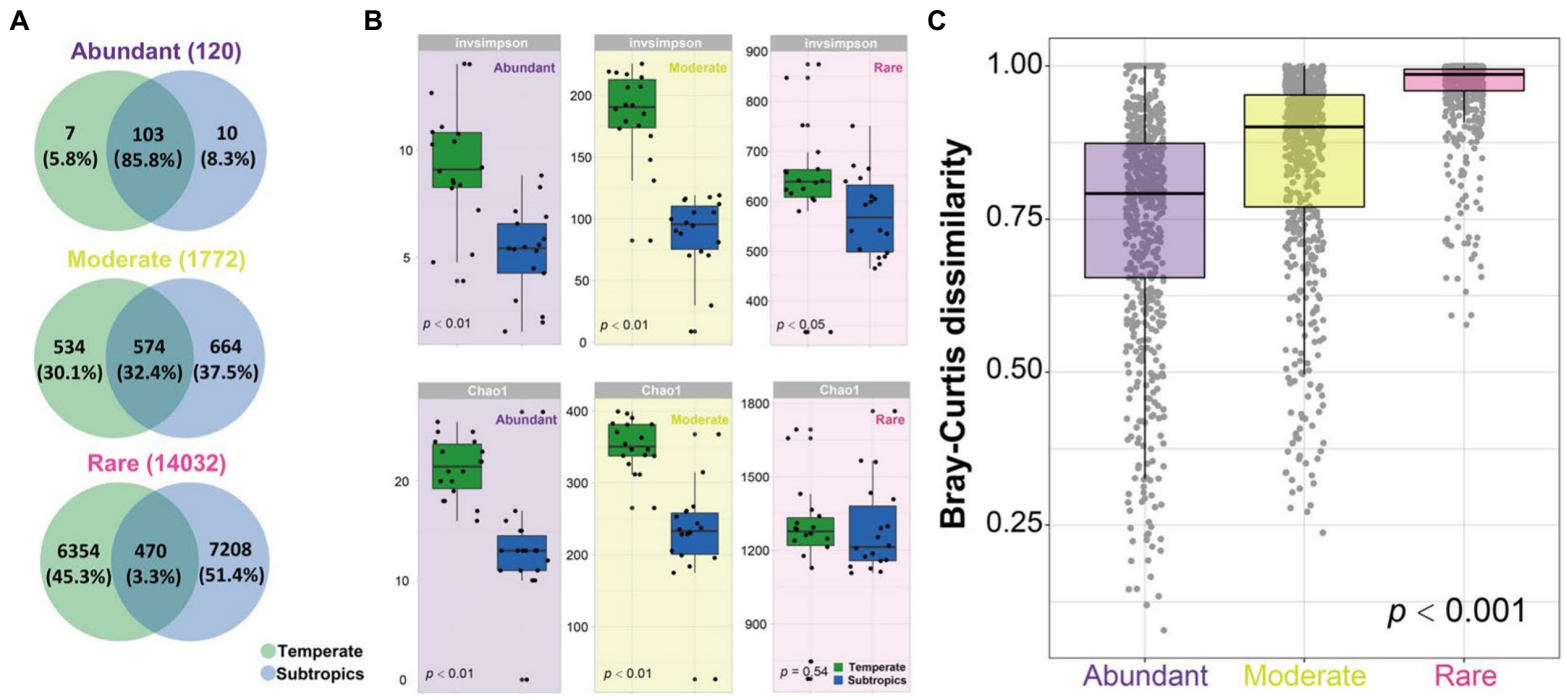
**(A)** Venn diagram showing the number of OTUs (120 abundant OTUs, 1,772 moderate OTUs, and 14,032 rare OTUs) that are unique and shared between two groups. **(B)** The alpha diversity of abundant, moderate, rare subcommunities in temperate and subtropics. **(C)** The boxplot shows community dissimilarities of abundant, moderate, and rare taxa.

The Venn diagram showed that most of the abundant OTUs (85.8%) were shared in the temperate and subtropical salt marshes, followed by moderate OTUs (32.4%), only a few rare OTUs (3.3%) were shared in indifferent groups (Figure 3A). Species richness (Chao1 index) of abundant and moderate taxa was significantly different in temperate and subtropical salt marshes (*p* < 0.01), while the Chao1 index of rare taxa was not significantly different between the two groups (*p* = 0.05). There were significant differences in the InvSimpson index of abundant (*p* < 0.01), moderate (*p* < 0.01), and rare (*p* < 0.05) taxa between the two groups (Figure 3B). The biggest differences were observed in the diversity of moderate taxa between the two groups (Figure 3B). Rare taxa had the highest beta diversity, followed by moderate taxa, while abundant taxa had the lowest beta diversity (Figure 3C).

### 3.3. Network analysis

**Figure 4:**
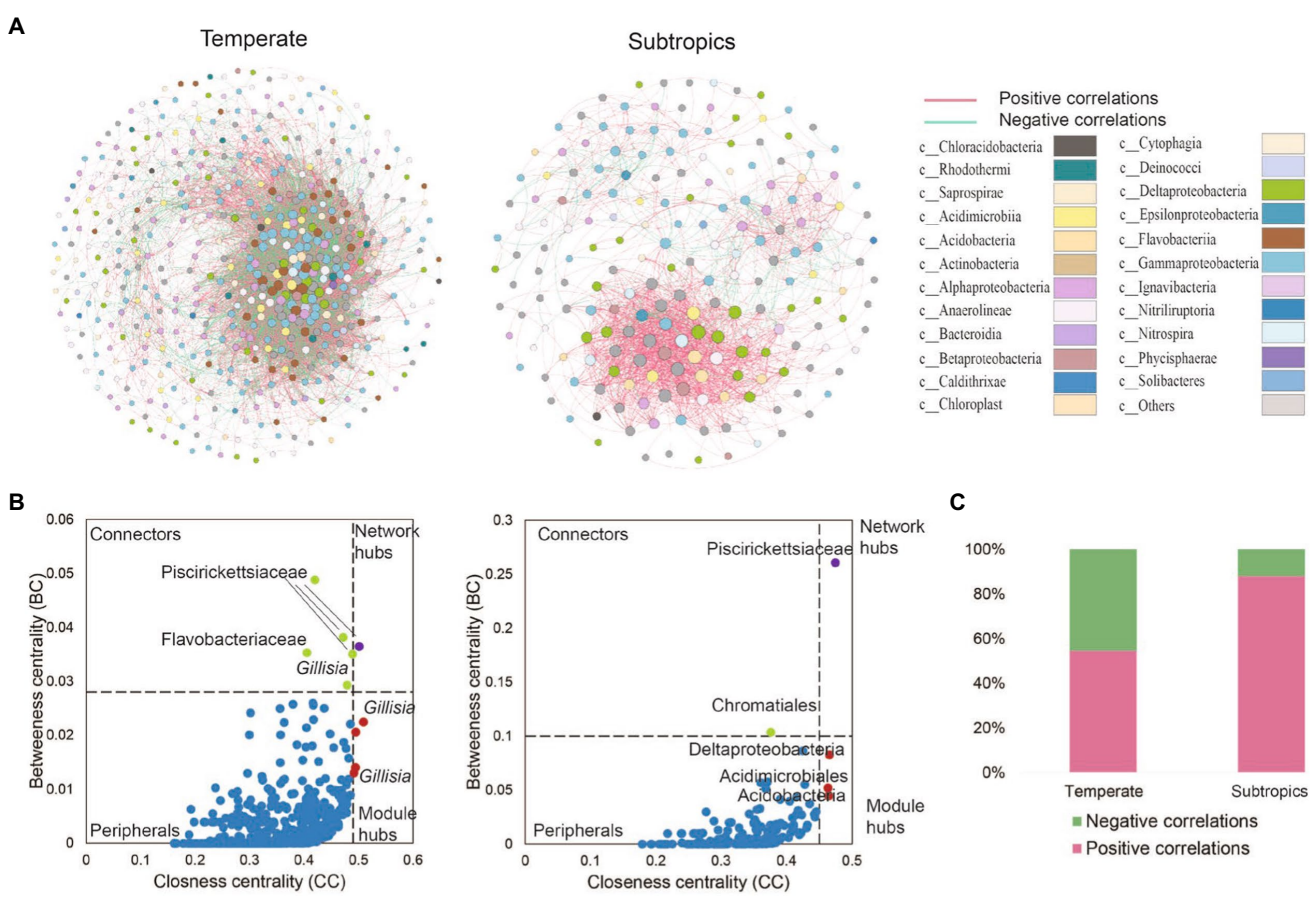
**(A)** The co-existence networks of bacterial co-occurrence patterns and **(B)** keystone hubs in different climates zones. Keystone hubs were visualized based on betweenness centrality (BC) and closeness centrality (CC) in the network of different treatments. In the temperate network, the module hubs were defined as BC < 0.028 and CC > 0.49, the network hubs as BC > 0.028 and CC > 0.49, the connectors as BC > 0.028 and CC < 0.49, and the peripherals as BC < 0.028 and CC < 0.49. In a subtropical network, the module hubs were defined as BC < 0.028 and CC > 0.49, the network hubs as BC > 0.1 and CC > 0.45, the connectors as BC > 0.1 and CC < 0.45, and peripherals as BC < 0.10 and CC < 0.45. **(C)** The proportion of positive and negative correlations detected in temperate and subtropical salt marshes.

The co-existence networks demonstrated distinct bacterial community structures in temperate and subtropical coastal salt marshes rhizosphere soils (Figure 4). In temperate rhizosphere soils, the network captured 10,621 strong (*r* > 0.6) and significant (*p* < 0.01) associations (edges) among 507 OTUs (nodes), with 54.57% positive correlations and 45.43% negative correlations. While in subtropical soils, the network had 196 nodes and 1,533 edges (88.0% positive and 12.0% negative; Figures 4A,C). In the temperate network, microbes had more intense connections with each other than in the subtropical network (average degree 41.9 vs. 15.6); while the subtropical network had a better modular structure (modularity 0.43 larger than 0.25 in the temperate network; Table 3). Further topological analysis on the centrality (closeness and betweenness) revealed an interesting shift of microbial hubs from temperate to subtropical habitats (Figure 4B). In the temperate network, most bacterial nodes within our ecological network (98%) were classified as peripherals, five nodes as connectors hubs, four nodes as module hubs, and only one node as a network hub. In the subtropical network, 97.5% of nodes were classified as peripherals, one node as a connector’s hub, three nodes as module hubs, and one node as a network hub. However, in both networks, the network hub, even though are different OTUs, remained in the same lineage, i.e., members of *Piscirickettsiaceae*. This indicates that *Piscirickettsiaceae* species might be a potential core taxon in structuring the rhizosphere microbial community in coastal salt marsh habitat, regardless of the location.

**Table 3:** Topological properties of the ecological networks in comparison to random networks.

	Temperate	Subtropics
Number of nodes	507	196
Number of edges	10,621	1,533
Avg. path length	2.952	3.154
Avg. clustering coefficient	0.582	0.615
Modularity	0.249	0.430

### 3.4. Effect of environmental factors

**Figure 5:**
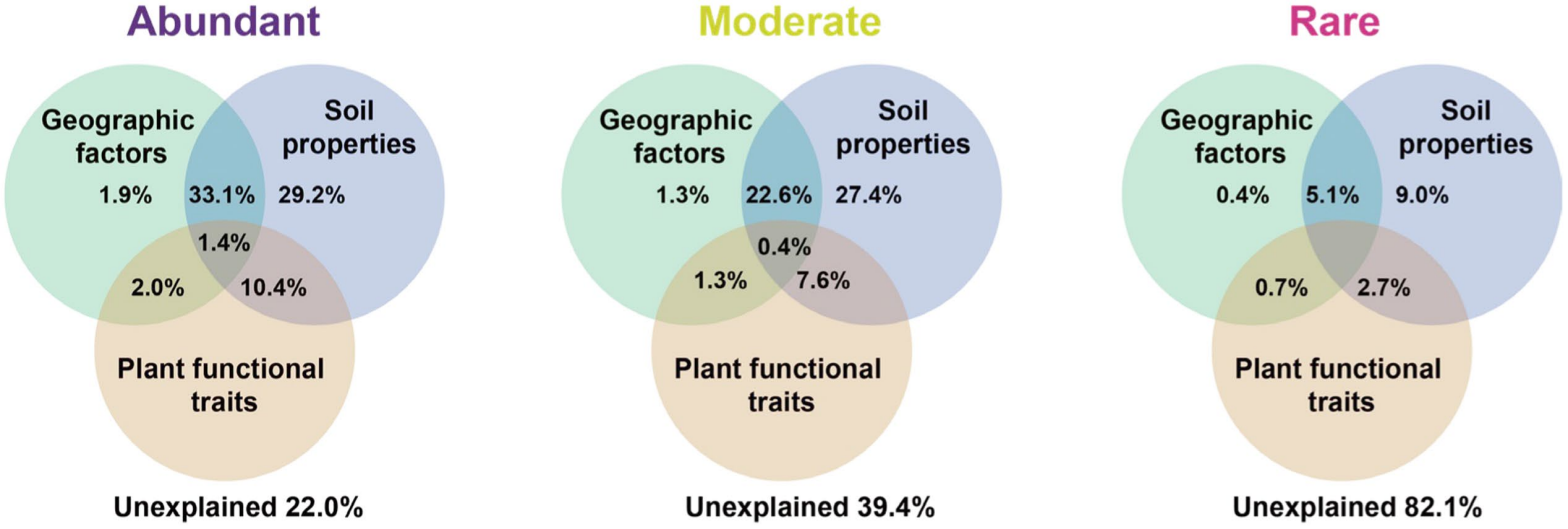
Variation partitioning analysis (VPA) showing the effect of geographic factors, plant functional traits, and soil properties variables on the community composition of the bacteria. Values indicate the percentage of variation explained by each section.

**Figure 6:**
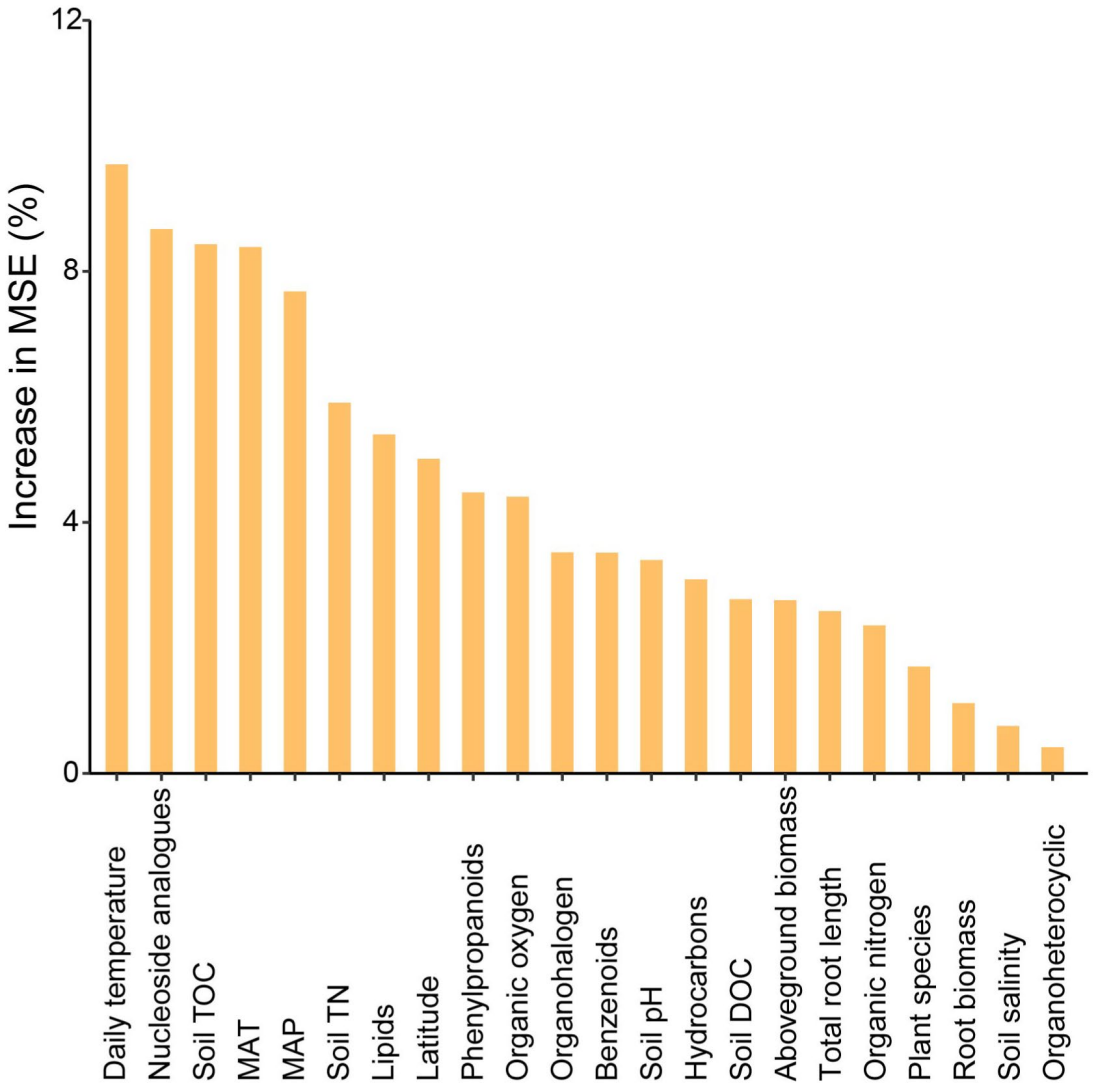
Main predictors of bacterial richness by random forest analysis. The figure shows the random forest results with the main predictor importance (% of increase of MSE) of environmental drivers on bacterial richness.

Variation partitioning analysis revealed that the relative contribution of soil properties (chemical properties and metabolites) on different bacterial groups was higher than geographic factors or plant functional traits. The explained proportion of environmental factors in abundant and moderate taxa was much higher than that in rare taxa. Notably, much of the variation (82.1%) in rare taxa was not explained by geographical factors, soil properties, or plant functional traits (Figure 5). Furthermore, the predictors of bacterial richness were identified *via* random forest modeling, and the result indicated that the daily temperature, nucleoside analogs, and soil TOC were strong predictors for bacterial richness (Figure 6). Interestingly, plant species had a very limited effect on bacterial richness.

## 4. Discussion

### 4.1. The differences in environmental factors and bacterial diversity in different climatic regions

There were significant differences in soil chemical properties, soil metabolites, and plant functional traits between the two climatic zones. Temperate salt marsh had higher soil nutrients (TOC, DOC, TN, soluble sugars, and organic acids) and lower soil pH. Because of the lower mean annual temperature (MAT), the decomposition rates were slower in temperate, where soil stored large quantities of organic matter. These organic matters such as organic acids decreased the soil pH. More metabolites showed significantly higher expression in subtropical regions, especially nucleoside analogs (deoxyinosine analogs and deoxycytidine analogs) and pesticides (tricyclazole and cordycepin), which probably come from cities or villages. The aboveground biomass was significantly higher in the subtropical salt marsh, while root biomasses showed no significant differences between the temperate and subtropical salt marshes. In our study, the MAT in the temperate salt marsh was significantly lower than that in the subtropical salt marsh. A global study has supported greater biomass distribution in roots and less in stems and foliage in colder environments at high latitudes (Reich et al., 2014). There was stronger cold stress in the temperate salt marsh, and plants in the salt marsh allocated more biomass to roots under stress conditions. By contrast, plants allocated more biomass to vegetative organs (stems and leaves) in the subtropical salt marsh (Reich et al., 2014).

### 4.2. Diversity patterns and keystone species of abundant, moderate, and rare bacteria in different climatic zones

Unlike the typical latitudinal diversity gradient (LDG), which means that species diversity is higher at lower latitudes (O’Hara et al., 2017). The alpha diversity of the bacterial community in temperate salt marsh soil was significantly higher than that in the subtropical zone, which was consistent with the results previously published (Bahram et al., 2018). The dominant phyla in the temperate and subtropical salt marshes were Proteobacteria and Bacteroidetes, and the relative abundance of these phyla showed no significant differences among abundant, moderate, and rare taxa (Figure 2C; Supplementary Figure S2). It has been reported that Proteobacteria, Bacteroidetes, and Actinobacteria were dominant groups in the coastal ocean (Campbell et al., 2011). Our study showed that abundant OTUs were shared among the different climatic zones (Figure 3A). Similar results have been reported in many studies. For example, a study in Hangzhou Bay showed that the most abundant bacterial taxa were shared across three transects from the water–land junction (Du et al., 2020). In particular, in some studies, all abundant taxa were shared in all samples (Liu et al., 2017; Mo et al., 2018). A marked difference in Bray–Curtis dissimilarity of abundant, moderate, and rare bacterial subcommunities indicated that the rare taxa were much more sensitive to changes in different climatic zones while the abundant taxa were more stable. Thus far, many studies have reported that the relative contributions of abundant and rare taxa to the stability of microbial communities were complicated (Jiao et al., 2019; Liang et al., 2020; Nyirabuhoro et al., 2021; Xiong et al., 2021). In agricultural soils, the abundant rather than rare bacterial taxa maintained microbiomes under environmental stress (Jiao et al., 2019). One possible explanation for this is that the abundant taxa might disperse easily because more individuals can potentially be involved in dispersal (Liu et al., 2015), while the higher dissimilarity of rare taxa may result from the limited dispersal, environmental selection, or drift for the rare species (Oono et al., 2017). Another possible explanation is that abundant taxa might have a wider niche breadth which might lead to higher resilience and resistance to environmental disturbances (Jiao et al., 2019; Yang Y. et al., 2022).

Network hubs, module hubs, and connectors were considered to play important roles in the stability and resistance of microbial communities (Tylianakis and Morris, 2017). Piscirickettsiaceae occupied the most nodes in the two networks. Most of the species from Piscirickettsiaceae are halotolerant or halophilic, and the family Piscirickettsiaceae includes important fish pathogens, which might be present in the coastal wetland (Ding et al., 2017). In temperate networks, *Gillisia* was determined as the module hub. It has been reported that *Gillisia was* significantly correlated with polycyclic aromatic hydrocarbons (PAHs), and *Gillisia* played a crucial role in the cycling of sulfur compounds in oil spills (Zhang et al., 2020). This is to be expected as the second (Shengli oil field) and the ninth largest oil field (Liaohe oil field) and located at the Yellow River Delta and Liaohe River estuary, respectively. Family Saprospiraceae are likely important in the breakdown of complex organic compounds in the environment (Lin et al., 2020). Some hubs were unclassified, suggesting that some bacteria uncovered from the salt marsh had not previously been identified, and they played an important role in bacterial networks.

The bacterial network became more complex from subtropics to temperate. One explanation may be attributed to higher organic carbon accumulation in temperate zones, which leads to a higher bacterial richness and a more complex network. Another reason may be due to the anthropogenic activities in cities and villages near sampling sites in a subtropical region, for instance, we observed more nucleoside analogs and pesticides in the subtropical salt marsh, which might be antibiotic drugs from cities. In addition, in the subtropical salt marsh, higher air temperature and solar radiation in summer likely reduce the activity of bacteria, and this was consistent with the observation of a higher effect of temperature on bacterial richness (Figure 6). These factors may lead to a simpler microbial network in the subtropical salt marsh. More negative correlations represent stronger competition, the higher bacterial richness could mean that more bacteria were competing for the same resource. We propose the high richness of bacteria in temperate salt marsh may lead to competition between the bacteria for the more efficient use of substrates.

### 4.3. Drivers of abundant, moderate, and rare bacterial subcommunities

In addition, the factors driving beta diversity (variation in community composition) of abundant, moderate, and rare subcommunities were also tested. Our study observed the importance of soil properties and soil metabolite for bacterial beta-diversity was observed in abundant, moderate, and rare bacterial subcommunities (Figure 5). Similar to previous studies, we found that abundant, moderate, and rare bacterial groups show different responses to environmental changes (Logares et al., 2013; Jiao et al., 2019). Abundant and moderate sub-communities were driven by identifiable environmental factors, while these abiotic and biotic factors can only account for a minor part of the variation in rare taxa. Many studies of community assembly have demonstrated that rare subcommunity was influenced by stochastic factors, while abundant taxa were mostly governed by environmental factors (Jiao et al., 2017). In this study, daily temperature, nucleoside analogs, and soil TOC exhibited a larger impact on bacterial richness (Figure 6). A possible explanation for this is that temperature variation could affect microbial biodiversity through a variety of mechanisms. A study in a forest ecosystem showed that the temperature is a primary driver in shaping soil microbial community in the forest soils (Zhou et al., 2016). In the subtropical summer, the temperature may increase to more than 40°C, higher air temperature and solar radiation in summer likely reduce bacterial activity and stability (Schultz-Fademrecht et al., 2008). Some nucleoside analogs have great potential to be purposed as antibiotics (Serpi et al., 2016), which are a large group of microbial natural products derived from nucleosides (Niu et al., 2019). Because of the lower average annual temperature, the soil TOC that accumulated from the remains of plants and animals in the temperate salt marsh was higher. These soil organic matters provide more nutrient resources for bacteria in summer.

## 5. Conclusion

Taken together, we showed that higher bacterial alpha diversity, more complex network structure, and more negative connections can be observed in the temperate salt marsh. These results implied that the bacterial community in the temperate salt marsh was more diverse and abundant than in the subtropical salt marsh in summer. Soil properties (chemical properties) and root exudates (metabolites) had the greatest influence on the bacterial community of salt marsh, especially for abundant and moderate taxa. However, 82.1% of the changes in rare species could not be explained by clearly identifiable factors, which might be explained by random factors such as drift. Our results can be beneficial for policymakers and salt marsh managers on the ecological services of coastal wetlands.

## Data availability statement

The datasets presented in this study can be found in online repositories. The names of the repository/repositories and accession number(s) can be found at: https://www.ncbi.nlm.nih.gov/, PRJNA914070.

## Author contributions

RW: investigation and writing—original draft. LC: methodology, writing—review and editing, supervision, and validation. JL: methodology and writing—review and editing. WL: supervision. All authors contributed to the article and approved the submitted version.

## Funding

This study was funded by the Fundamental Research Funds of CAF (CAFYBB2020QB008).

## Conflict of interest

The authors declare that the research was conducted in the absence of any commercial or financial relationships that could be construed as a potential conflict of interest.

## Publisher’s note

All claims expressed in this article are solely those of the authors and do not necessarily represent those of their affiliated organizations, or those of the publisher, the editors and the reviewers. Any product that may be evaluated in this article, or claim that may be made by its manufacturer, is not guaranteed or endorsed by the publisher.

## Supplementary material

The Supplementary material for this article can be found online at: https://www.frontiersin.org/articles/10.3389/fmicb.2023.1127958/full#supplementary-material

SUPPLEMENTARY FIGURE S1 The geographical location of the sampling sites.

SUPPLEMENTARY FIGURE S2 Percentage of relative abundance of sequences of abundant, moderate, rare bacterial taxa in salt marsh.
