# Supplementary figures and images for: Factors driving the halophyte rhizosphere bacterial communities in coastal salt marshes
Source: Front Microbiol. 2023 Feb 22;14:1127958. doi: 10.3389/fmicb.2023.1127958 (PMC9992437; doi:10.3389/fmicb.2023.1127958)

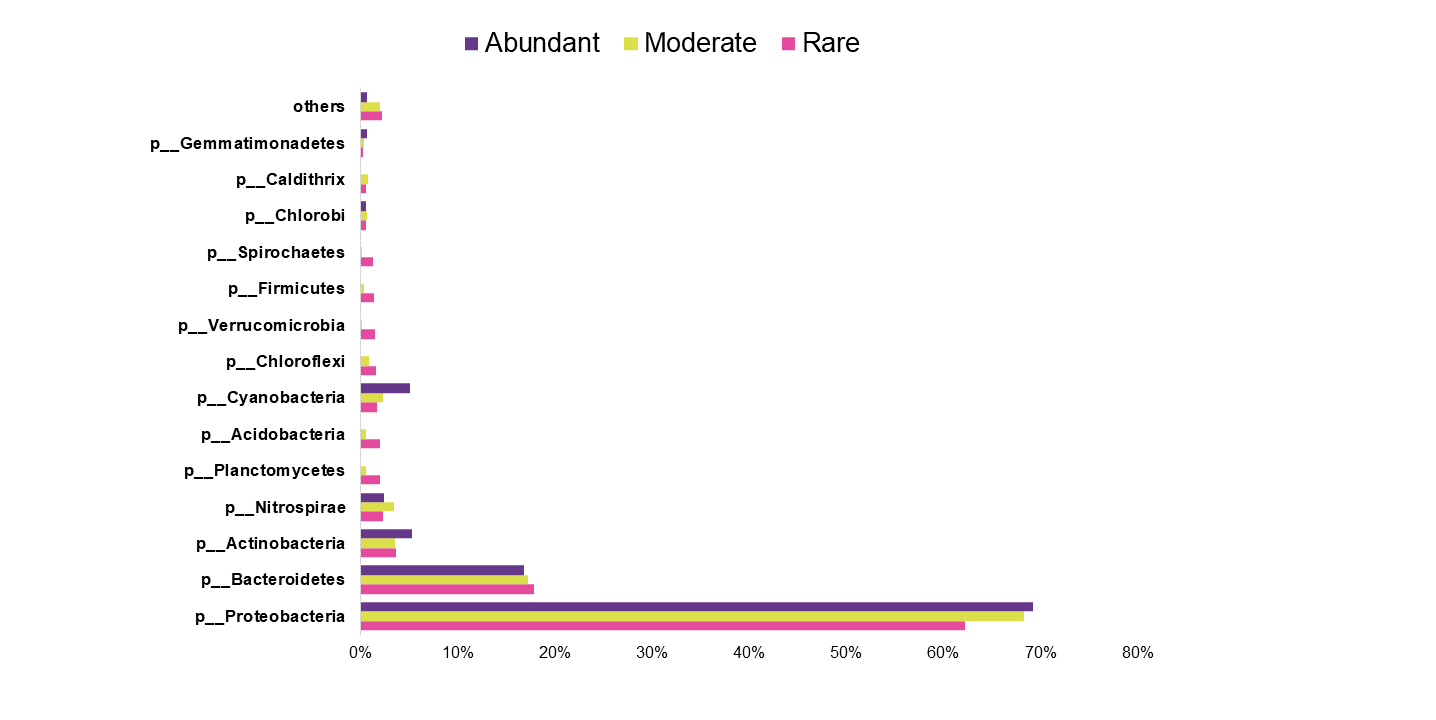

Supplement: SUPPLEMENTARY FIGURE S2 — Percentage of relative abundance of sequences of abundant, moderate, rare bacterial taxa in salt marsh. [file Image_2.PNG]
